# Crossing knowledge boundaries: health care providers’ perceptions and experiences of what is important to achieve more person-centered patient pathways for older people

**DOI:** 10.1186/s12913-021-06312-8

**Published:** 2021-04-07

**Authors:** Cecilie Fromholt Olsen, Astrid Bergland, Asta Bye, Jonas Debesay, Anne G. Langaas

**Affiliations:** 1 Department of Physiotherapy, Faculty of Health Sciences, OsloMet- Oslo Metropolitan University, Oslo, Norway; 2 Regional Advisory Unit in Palliative Care, Department of Oncology, Oslo University Hospital, Oslo, Norway; 3 Department of Nursing and Health Promotion, Faculty of Health Sciences, OsloMet- Oslo Metropolitan University, Oslo, Norway

**Keywords:** Older people, Quality improvement, Health care providers, Home health care services, Transitional care, Patient pathways, Person- and patient-centered care, Qualitative research

## Abstract

**Background:**

Improving the transitional care of older people, especially hospital-to-home transitions, is a salient concern worldwide. Current research in the field highlights person-centered care as crucial; however, how to implement and enact this ideal in practice and thus achieve more person-centered patient pathways remains unclear. The aim of this study was to explore health care providers’ (HCPs’) perceptions and experiences of what is important to achieve more person-centered patient pathways for older people.

**Methods:**

This was a qualitative study. We performed individual semistructured interviews with 20 HCPs who participated in a Norwegian quality improvement collaborative. In addition, participant observation of 22 meetings in the quality improvement collaborative was performed.

**Results:**

A thematic analysis resulted in five themes which outline central elements of the HCPs’ perceptions and experiences relevant to achieving more person-centered patient pathways: 1) Finding common ground through the mapping of the patient journey; 2) the importance of understanding the whole patient pathway; 3) the significance of getting to know the older patient; 4) the key role of home care providers in the patient pathway; and 5) ambiguity toward checklists and practice implementation.

**Conclusions:**

The findings can assist stakeholders in understanding factors important to practicing person-centered transitional care for older people. Through collaborative knowledge sharing the participants developed a more shared understanding of how to achieve person-centered patient pathways. The importance of assuming a shared responsibility and a more holistic understanding of the patient pathway by merging different *ways of knowing* was highlighted. Checklists incorporating the *What matters to you?* question and the mapping of the patient journey were important tools enabling the crossing of knowledge boundaries both between HCPs and between HCPs and the older patients. Home care providers were perceived to have important knowledge relevant to providing more person-centered patient pathways implying a central role for them as knowledge brokers during the patient’s journey. The study draws attention to the benefits of focusing on the older patients’ way of knowing the patient pathway as well as to placing *what matters* to the older patient at the heart of transitional care.

## Background

Older adults often have multiple chronic diseases and, thus, complex and unpredictable pathways within the healthcare system [1, 2]; they require treatment and care from a wide range of health care providers (HCPs) and services concurrently and frequently make transitions between primary and secondary health care services [3, 4]. Transitional care is a broad term for care interventions that promote the safe and timely transfer of patients between levels of care and across settings [5–7]. It encompasses a complex set of interventions and environments to ensure interaction among HCPs and with the patient and informal caregiver as the patient moves through the care system [7, 8]. Previous research has shown that these transitions pose significant challenges and risks for older patients [8–11]. Adverse events such as medication errors, falls, and infections during and immediately after hospital discharge are common [11], the reasons for which range from patient complexity, low quality of assessing the older patients’ needs and involving them in care, communication errors, and organizational and cultural boundaries [1, 12–15].

Overall, current literature reviews present a large variety of interventions including single and multimodal strategies such as care pathways and transitional care models to improve transitional care for older people [8, 16–20]. The effectiveness of such interventions appears to be highly context dependent; however, some elements seem to be more important than others [13, 14, 16–18, 20–22]. Pertaining to hospital to home transitions for older people, central elements seem to be well-planned hospital discharge, communication/information exchange, self-management education as well as adequate follow up by primary care when the patient is at home focused on the monitoring and prevention of decline in health status [1, 2, 6, 14, 16, 17, 19, 22, 23]. Moreover, to achieve improvement in transitional care for older people the aspect of person-centered care is gaining increased attention [1, 12, 13, 15]. Nonetheless, when reviewing previous transitional care interventions, there tends to be a stronger focus on improving hospital readmission rates, cost-effectiveness and communication among HCPs, and less on improving the person-centered aspects of transitional care for older people [13, 14, 21, 24], which is the focus of the current study. Involvement of the patient and informal caregiver and their unique perspective of the patient pathway has the potential to play an important role in creating and sustaining quality and safety during transitions [7, 12, 15, 18, 24–27]. Older people’s involvement during care transitions may be particularly impeded by disease-related factors such as pain and reduced cognitive processing, as well as the many care events happening within a short period in unfamiliar environments [12, 28]. Interventions may claim to adhere to person-centered principles, but it is unclear how this is actually enacted in practice [1, 15, 29].

### Conceptual framework

Person- or patient-centered care (PCC) are slightly different concepts but are often used synonymously [30]. According to a recent review [30], the common themes for both concepts are empathy, respect, engagement, relationship, communication, shared decision making, holistic care, individualized focus, and coordinated care. In the context of transitional care for older people, the triad of HCP, patient, and informal caregiver is essential for PCC [7, 31]. Furthermore, as a way of enacting PCC in practice, the concept of “knowing the patient” has been explored, mostly within the literature on nursing, but with arguable relevance to other HCPs. According to Radwin [32], *knowing the patient* entails a complex process whereby the HCP acquires information about and an understanding of a specific patient as a unique individual; this consists of getting to know the experiences, behaviors, and patterns of responses of an individual patient [33]. This is expected to enhance clinical decision making, the selection of optimal interventions, and patient outcomes. It is emphasized that time and involvement on behalf of the HCP is needed to get to know and build a relationship with the patient so that a HCP can make good care decisions [32, 33].

A *patient pathway* is defined by Norwegian health authorities as “a holistic, coherent description of one or several patients’ contacts with different parts of the health care system during a period with disease” [34]. Thus, a patient pathway is not a standardized model of care, such as a CP. A CP is defined as a complex intervention for the mutual decision making and organization of care processes for a well-defined group of patients during a well-defined period [35]. CPs are depicted as models or flow charts that map out chronological key activities in a healthcare process, and they are often outlined in procedures or checklists [36, 37]. A central aspect of a CP is the standardization of care processes; however, a CP is also seen as an opportunity to focus on person-centered care so that care is organized around patient journeys, not the organizational units of the health care system [35]. In Norway, several types of CPs are developed for different patient groups and in different settings. Many CPs are created nationally and adapted locally [2, 38, 39]. Patient journey mapping is a salient feature of CP development that aims at making the process more patient centered. Patient journey mapping refers to the mapping and visualization of patient journeys as experienced and told by the patients. It stands apart from the service providers’ perspective, and the goal is to increase the awareness of the patient perspective and keep the quality improvement process person-centered [40]. Hansen et al. [25] posit that the concept of a person-centered patient pathway necessitates a focus on the patient pathway as something more than the purely clinical journey; both *health* and *life* events should be considered [1, 25, 41, 42].

PCC has proven challenging to implement within single-care settings [1, 15, 43, 44]. It may be even more challenging for HCPs to practice when older vulnerable patients transition between services [1, 15, 24, 26, 45] and the evidence base for this practice remains unclear [1, 15]. Central to understanding these challenges is the considerable complexity of the transitional care context, especially the presence of several types of boundaries [8]. Examples of such boundaries are spatial and organizational boundaries, cultural boundaries, and knowledge boundaries [8]. Knowledge boundaries relate to the different ways in which actors and social groups give meaning to patient care, quality, and patient pathways and where different views underpin fragmentation [8]. Given our focus on PCC in this study, it is essential to understand that knowledge boundaries do not only exist between collaborating HCPs, but also between the HCPs and their patients [46].

### A quality improvement collaborative to achieve person-centered patient pathways

The successful crossing of knowledge boundaries seems essential to improving quality and safety in transitions [8, 18, 47–50]. Therefore recent reviews on safety and quality in care transitions [1, 12, 18, 51] call for more research on person-centered knowledge sharing interventions that emphasize relationships and interaction to overcome boundaries. In this study, we followed HCPs’ work in a Norwegian quality improvement collaborative (QIC) whose goal was to achieve more person-centered patient pathways for older people. Norwegian policy endorses person-centered care and coherent patient pathways for older people [52, 53]. Therefore Norwegian health authorities initiated a national QIC called “learning networks for good patient pathways for older and chronically ill people” to stimulate and support HCPs in implementing this policy [54]. A QIC is a short-term learning system involving multidisciplinary teams from various health care settings that come together over several months to improve their provision of care; here, knowledge sharing is a central aim [55] and knowledge boundaries become relevant. The current collaborative engaged HCPs from primary care and a local hospital involved in the transitional care of older and chronically ill people in a large municipality. The participating HCPs were introduced to two measures to guide their improvement efforts: 1) a person-centered turn from asking, “What is the matter with you?” to asking, “What matters to you?” (WMTY) [54, 56] and 2) local tailoring of “Patient Trajectory for Home-dwelling elders” (PaTH), a patient-centered and checklist-based CP that was developed in Norway for the care of older people in need of follow-up by primary and home care after hospital discharge [37, 57, 58].

### Aim

Whilst patient experiences are arguably the main focus of inquiry in person-centered care [12], HCPs facilitate and enact patient pathways and play a crucial role in involving older people and their informal caregiver to assure high quality transitional care [1, 16, 24]. Understanding HCPs’ perspectives and experiences is therefore important [12]. The aim of the present study was therefore to explore HCPs’ perceptions and experiences of what is important to achieve more person-centered patient pathways for older people; this was by following the work in the above described QIC. The article contributes important knowledge that could be useful for the future quality improvement of transitional care for older people.

## Methods

The present article is based on data from a large qualitative study of HCPs’ understandings and experiences of working towards more person-centered patient pathways for older people [59]. A previous publication from this larger study presents the HCPs’ experiences and perceptions of the WMTY approach [56]. Whereas this article focuses on a different research question, namely; *According to the HCPs’ perceptions and experiences, what is important to achieve more person-centered patient pathways for older people?* We triangulated methods and data [60, 61] from semistructured interviews of 20 HCPs and 3 key persons as well as participant observations of 22 meetings, and central documents in the QIC. The current study was informed by a constructivist epistemology [62], and the article follows the consolidated criteria for reporting qualitative research (COREQ) [63].

### Research context

In Norway, healthcare is universally accessible and primarily publicly funded. There is a two-level model of care where specialist care and hospitals are owned by state authorities and primary care is run by the municipalities [64]. General practitioners (GPs) are self-employed and are medically responsible for home-dwelling older people [64, 65]. The chosen QIC took place in a municipality with a complex organization comprising several local hospitals, home care service organizations, intermediate care services, and numerous GPs. Because of the size and organizational complexity of the municipality, only a select sample of the total primary care institutions (intermediate rehabilitation units and acute municipal care), GPs, and home-service organizations—here with their local hospital—participated in the current QIC. For the same reason, a separate project team was established to facilitate the local improvement work. The plan was to scale up the implementation of the locally tailored CP to the whole municipality after the initial 18-month period of the QIC.

The QIC was based on the model called *The Breakthrough Series,* which was developed by the Institute of Healthcare Improvement in Boston [55, 66, 67]. The current collaborative included around 90 participants who met over a period of 18 months and comprised local improvement teams of mainly front-line workers from different professions, each with a designated team leader. The participants met at four communal *learning sessions* that consisted of expert lectures and group discussions. In between these, the participants worked within their teams to implement their chosen local improvement measures in a bottom-up manner. Team leader meetings were held regularly to keep track of the work and share knowledge and to locally tailor the PaTH. The PaTH serves as a framework when Norwegian municipalities develop CPs for older and chronically ill people [39, 68]. Based on qualitative studies [2, 57], it was established that disease-specific CPs were not feasible in primary care. Thus, the PaTH was designed as a generic and function-based CP that aimed to be patient-centered by incorporating the WMTY question into the checklists [2, 37, 45]. As part of the support to include the patient perspective in care, the QIC encouraged interviews with older patients concerning their experiences of their patient journey, the visual mapping of patient journeys and inclusion of user representatives in the improvement teams.

The current research project was independent of the QIC. We adopted an exploratory approach instead of an evaluative approach. Hence, the study’s aim was not to describe the whole range of different topics discussed in the QIC but instead to focus on the participants’ meaning-making as it related to person-centered patient pathways.

### Recruitment

The inclusion criteria for participating in the present study were to be an HCP and involved in the activities of the relevant QIC. The QIC administrators gave permission and access to the field. Prior to the first learning session, an email containing project information and written consent forms was sent to the QIC participants. The consent forms contained optional check boxes for observation and interviews, respectively. Recruitment to the research was independent of the QIC recruitment. It continued throughout the collaborative period (18 months) because there was a degree of turnover in the improvement teams. In total 134 persons consented to observation, of these 77 persons also consented to individual interviews. Among the 77, a purposively collected sample [69] was chosen based on the need for variation and information richness [70]. Improvement team leaders were chosen because they were well-informed regarding the topics of interest. Participants from all settings were selected; however, we prioritized workers from primary care, especially home care, to reflect a focus on follow-up in home care in the PaTH and the QIC. We assessed that there was enough information power [70] and stopped recruiting for interviews after 20 HCP interviews, which was close to the sample size we had estimated. In addition to interviewing 20 HCPs, we interviewed three key persons who were leaders or administrators of the QIC to gain an understanding of the contextual influences of the QIC.

### Ethical considerations

The research project was preapproved by the Norwegian Center for Research Data (Reg No. 54438) which was the only relevant review board since the study did not involve patients. The study was performed according to the Declaration of Helsinki [71]. Informed swritten consent was obtained from the participants. The participants were assured that participating in the research was voluntary and that choice to participate would not affect their participation in the QIC. They were informed about their right to abstain from participation or withdraw from the study and that data would be handled confidentially. Finally, no one withdrew from the study.

### Data collection

Data collection spanned the QIC period of 18 months between 2017 and 2019. The first author conducted the interviews and observations. A thematic interview guide that was published with the first article from this study [56] was used; this guide was based on exploring HCPs’ experiences and understandings of how to improve older patients’ pathways. For example, the researchers inquired about the perceptions of and experiences with asking WMTY and involving older people in care, the perceptions of a good and bad patient pathway for an older person, the experiences and challenges with current patient pathways, and experiences with tailoring the PaTH. Central documents, such as the PaTH and WMTY tools, were often used in the interviews to facilitate reflection. A flexible approach [69] was chosen to allow the participants to reflect on and talk about the improvements they were working on during the process and to clarify any issues that might come up during the observations. Thus, the interviews and observations complemented each other to ensure the credibility of findings [60, 69]. The interviews were conducted in the participants’ respective workplaces. They lasted from 60 to 90 min, were digitally recorded, and were transcribed verbatim.

The first author was a moderate participant observer [72] at six local improvement team meetings (*n* = 3–10), 13 team leader meetings (*n* = 10–18), and three administrators’ meetings (*n* = 5–7) (61.5 h in total). Several key persons with expertise in relevant areas, such as electronic health record systems and care models, often attended the QIC meetings and provided support to the HCPs in the process. The meetings were held in the respective workplace environments and in the administrators’ localities and lasted from one to seven hours. The researcher was also present at the learning sessions, which took place in conference venues.

During observations of the meetings, the researcher sat together with the participants and engaged in appropriate actions and small talk but did not take part in the participants’ discussions. This was in line with what Spradley [72] describes as moderate participation, where the researcher has to balance the role of being an outsider and insider. Features of the setting, actions performed, and the tools used were written down, along with what the participants discussed. The participants knew the professional background of the researcher in that she had previously worked in some settings represented in the QIC. They were also informed about the purpose of the study and the independence of the research project from the QIC. Because the researcher met with some participants frequently, a relationship was built, and this facilitated data collection.

### Data analysis

Relevant QIC documents were scrutinized and served to clarify and validate the other data. We followed a thematic analysis approach in six interrelated steps as described by Braun and Clarke [73] to identify meaningful patterns of information in the data. Primarily, the codes and themes were inductively developed (see Table 1). We focused specifically on the parts of the data that revealed something important regarding the current research question. Codes that revealed similar aspects of the data were grouped into preliminary themes, which were checked for consistency and variability within and across interviews and observations. The interpretation of these themes involved a process of reading and writing, as well as reference to relevant literature and consultations among authors, eventually resulting in five interrelated themes. Observation data and interview data contributed equally to the analysis; however, quotes were mostly chosen from the interview data based on their readability. NVivo software [74] was used to manage the analysis process. An analytical memo was kept by the first author.

**Table 1:** Example of the analysis process; codes, preliminary- and final themes

Examples of codes	Preliminary themes	Final theme
Patient journey- new discoveries Trusting each other more Checklists clarify responsibility Checklists increase predictability Learning about and from others Getting to know each other From the patient’s point of view Wanting the best for the patient Colleagues around the user Working together for the sake of the user Common understanding-common goal WMTY- from random to systematic WMTY-Working *with*, not for, the user	Formal and informal practice is revealed New discoveries about the patients, new discoveries about each other Negotiating checklists to improve the patient journey What we have in common is the patients Facilitating a new common understanding of the patient journeys	Finding common ground through the mapping of the patient journey
Thinking in more holistic terms Understanding the system A shared responsibility A more holistic approach “Patient pathway” triggers person-centeredness Task orientation Thinking ahead	From task orientation to more person-centered care Not just here and now, not just me Patient has a past present and a future Expanded knowledge about the pathway	The importance of understanding the whole patient pathway
Finding someone who knows the patient Being knowledgeable and professional Home care knows them well Getting acquainted WMTY-same but different Safety for the patients Talking to informal caregiver Next of kin as resource More holistic information about the patient Being knowledgeable and professional Establishing a common language Focus on documentation content Signaling we know you	The home as the alpha and omega of the pathway Knowing the patient in different ways Seeing a different version of the patient	The significance of getting to know the older patient
It’s often too late Out of sight, out of mind Keeping them at home Focus on physical function at home Safety at home Focus on standardized assessment at home More proactive thinking	Home care nurse as a link between the before and after in the patient’s journey They know the patient and their home-situation best From task orientation to more comprehensive care Keeping older people safe at home	The key role of home care providers in the patient pathway
Skepticism towards the QIC work Checklists- not being used Checklists- not the whole solution Checklists- enthusiasm Checklist- skepticism Importance of anchoring with leaders Unpredictable pathways Overwhelming complexity Need for resources to implement	Checklists as universal remedy Unpredictable patient pathways within a more systematic frame The checklists are a symptom- not the whole solution From random to systematic care for the older patient Bottom- up is good but necessitates top-down support Resources are crucial for implementation	Ambiguity towards checklists and practice implementation

Regarding the researchers’ positions and preconceptions [69], the first author’s first hand experiences with the challenges and dilemmas of achieving person-centered patient pathways for older people influenced the study. For example, the choice of research question was both based on an identified knowledge gap and on personal interest. Also, the choice to highlight the role of home care providers was both based on the data and on prior work experiences of the usefulness of their competency. Even though all the authors have professional and research interests in the field of health science, there was a diversity among the authors’ backgrounds (nursing, anthropology, physiotherapy, and dietetics), which led to interesting discussions and enhanced reflexivity [75]. The experiences of the researchers of the present study within health care means that there were probably certain things that we were “blinded” to and took for granted; however, it also means that we were well positioned to understand the context of and perform the study [61, 76] (see Table 2 for more details).

**Table 2:** Sources of trustworthiness [60] in the current study

Quality dimension	Steps taken in the study to enhance the dimension
Credibility	Flexible complementary data collection approach Prolonged engagement with the field Prolonged engagement with the data Comprehensive description of methods Themes agreed upon through reflexive discussions among authors
Dependability	Last author validated first author’s coding Several authors engaged in analysis
Confirmability	Reflexivity Quotes, theory and previous research used to substantiate findings Analytical memo keeping
Transferability	Thorough description of context Quotes, theory and previous research used to substantiate findings

## Results

### Participants

The 20 interviewed HCPs, which included four men and 16 women (mean age: 43.9), were all chosen by their respective superiors to take part in the QIC activities. Seven of the participants were leaders of their local improvement team. Three of the HCPs did not currently work on the front line. Seventeen of the participants worked on the front line with patients, some with mixed administrative or educational tasks. Table 3 presents more information. To maintain anonymity, the participants are only referred to with participant number (according to appearance in the text) and as either working in home health care services or in an institution (comprising intermediate care institutions and hospital).

**Table 3:** Characteristics of the interviewed participants

Male/female	Profession	Age	Work setting	Time in current position	Years of education after high school
**4/16**	**Nurses 12 Physiotherapists 5 Medical doctor 1** **Nurse assistant 1 Occupational therapist 1**	**Mean 43.9 years** **Range 29–59 years**	**Home care 13 Hospital 2** **Intermediate care 5**	**Mean 5.1 years** **Range 6 months - 17 years**	**Mean 5.1 years** **Range 3–10 years**

### Themes

The following results are presented as five interrelated themes. In different ways, the themes are related to the HCPs’ perceptions of salient factors to achieve high quality person-centered transitional care for older people.

#### Finding common ground through the mapping of the patient journey

Overall, the participants emphasized that it was crucial for the QIC work to see the patient pathway from the older patient’s point of view. In line with CP development methods, mapping the patient journey, interviewing patients, and working with user representatives became important reflection tools. Regarding interviewing older patients, one said:

*We learnt a lot about how the user experienced it, it gave a couple of aha- experiences. I had not seen it from their perspective before.* (Participant 1, home health care services)

When working on the patient journeys, the participants identified improvement needs in their local work processes, as well as in work across settings. In particular, the WMTY question was discussed extensively. These activities seemed to facilitate new and shared ways of thinking about care and the participants seemed to find common ground. It became apparent to the participants that the assessment of *what mattered* to the patient as they moved through the system was random and unsystematic. Consequently, HCPs from all the different work settings in the QIC worked to get a system in place for how to engage in the WMTY approach. The participants highlighted that through the QIC work, they understood that they all cared about what mattered to the older patients. Thus, trust in collaborating partners to care for older patients increased.

Furthermore, the issues raised by the patient journey work were believed to at least be partially solved through the local tailoring of the PaTH checklists. The participants believed that by working more systematically both with the WMTY approach and with the content of the checklists, older patients’ experiences of their journeys would be improved. However, this was viewed as a big challenge. One participant said the following:

*Well, it requires a total reestablishment of everything, everything from reports to how things are put into words, the use of terms and, well, thinking holistically about people.* (Participant 2, home health care services)

Toward the end of the QIC work, the improvement team leaders from the different care settings initiated a negotiation process to locally tailor the PaTH checklists, here focusing on “what we need from you and what you need from us.” Hence, during the QIC process, the participants developed new understandings of their own and other collaborative partners’ work. Many important bottlenecks were discussed, and dilemmas and tensions were brought to the surface. The knowledge that they shared was both of a formal and informal character, and both were deemed important from the HCPs’ point of view. During the QIC, the participants learned both *what* other HCPs were doing and *why* they were doing it. A participant who was interviewed toward the end of the process said the following:

*So, just getting to know each other a bit better and getting a better understanding of the routines of the hospital. That we get a bit closer, that it is not just you and me, but that we are really colleagues working around the user. That we wish to accomplish the same things. I think that has been very useful. That we understand each other’s everyday life better.* (Participant 3, home health care services)

Hence, working together in the QIC to improve the patient journey seemed to give the participants a sense of common purpose and goals and mutual trust increased. During a meeting, a participant said: *What we have in common is the patients.* (Observation note, team leader meeting).

#### The importance of seeing the whole patient pathway

The increased focus on the patient journey seemed to enhance the participants’ awareness of the totality of the patients’ pathways from hospital discharge through the different intermediate care facilities and home care. Therefore, the participants emphasized that to make the patient pathways more person-centered, all HCPs in the care pathway had to assume a shared responsibility for the *whole* patient journey. Also, they must understand that their own daily work processes, although isolated in space and time, were part of a larger system of care around the older patient and their next of kin. Many of the participants expressed that this was a less-fragmented and more rewarding way of thinking and working. One participant said the following:

*Holistic, really, in the way that when I go to him [the user], I do not just do this and that and my tasks without thinking about what has been and what will be. You know, that I do not just do my things and get them over with.* (Participant 4, home health care services)

What the participants called a more *holistic* approach meant thinking beyond each HCP’s narrower perspective and seeing care as many interconnected steps that together made up the patient pathway. The ideal appeared as seeing care more in terms of an ongoing process with an overreaching goal rather than as time-limited tasks to be performed separately without an underlying purpose. To achieve good patient pathways, it was important to understand that the older patient followed their own unique journey with a past, present, and future, which also involved other professionals and other settings.

#### The significance of getting to know the older patient

The participants agreed on the importance of getting to know the older persons and their next of kin. This was perceived as important for the older patient’s sense of safety and trust in HCPs during the vulnerable phase of transitions. However, between the different settings, there were different understandings regarding what it meant *to get to know* the patient. It became clear that although the WMTY question signified patient involvement, it also meant different things in different settings and to different professionals. The differences between settings were discussed to a much larger degree than the differences between professionals.

It appeared important that the HCPs in the different settings needed to develop a shared assessment of the needs of the older person. In hospital and institutions, they often described a frail older person, mostly lying in their bed with hospital clothing and going through some worsening of a chronic condition and physical function. Getting to know the patient meant assessing their medical condition first and foremost, *then* focusing on physical function, and *then* asking what matters. One stated the following:

*We write a lot of electronic reports based on our experience of how we see the patient when he is admitted here. And then maybe we see a frail older person and we haven’t looked into how they are able to function at home. And then immediately we think “oh they need a nursing home placement, oh poor person.”* (Participant 5, institutional care)

The work in the QIC made them realize that they only had a momentary picture of the patient and, hence, could misunderstand or misinterpret the older patient’s ability to manage their life at home after discharge. There seemed to be enhanced awareness that there was more to know about these patients.

Workers from the home care services saw the older person in their own home environment. Getting to know the patient meant getting to know their family and life situation, which involved attaining more detailed knowledge of their daily habits, preferences, and ways of living. This was perceived to be easier when working in the home of the older person. HCPs from the home care sector expressed some concerns regarding the way older patients were assessed and treated in institutions. The home care workers were concerned that patients became “institutionalized” in the hospital and intermediate care settings. They were concerned that the patients were deprived of the possibility of trying to manage things on their own, leading to larger loss of function than necessary. Another participant who worked with patients in their home said the following about how HCPs should be thinking regarding older people’s patient pathways:

*So that already in specialist healthcare one starts to think that, well actually, this person should try to manage in the shower alone, try to handle the medicines, try to walk the stairs by themselves, do all those things as soon as possible because that is what the user is going back to.* (Participant 6, home health care services)

It was emphasized that when older patients returned home after institutional stays, they often managed better than what was anticipated by the HCPs in hospitals and intermediate care. Assessing *what matters,* which can be understood as motivation for self-care and independent function, was perceived to be easier when working in the older person’s home.

The importance of knowing the patient and patient’s journey to improve the patient pathways was also reflected in the participants’ perceptions of documentation, information exchange, and discharge communication. Thus, the ideal was that more of the situation around the patient be described, for example, how they usually managed at home and their relationship with their next of kin. Knowledge about the information needs of their colleagues in other settings in the pathway was deemed crucial for high quality transitional care. Establishing a common language was highlighted as important. This was believed to facilitate appearing as an informed unit toward the patient. One participant said the following:

*That the user doesn’t have to repeat herself at each new place, but feels like the people I relate to here, they communicate, they know who I am [ … ] that the user feels like when they ask me questions they ask them as if they already know me a little*. (Participant 7 – home health care services)

Hence, signaling to the patient “we know you” was seen as an important aspect of person-centered patient pathways.

#### The key role of home care providers in the patient pathway

Because of their particular knowledge about the older person in the older individual’s own context, it was discussed how the providers of home care services, especially nurses, should serve as a spokesperson for the older patient during transitions. This included following up on the patients during GP visits, institutional stays and care transitions. For example, one improvement measure discussed at the meetings was making a routine out of involving HCPs from home care services in the multidisciplinary family meetings at the intermediate care units. One home care worker who had experienced this said the following:

*The users are very happy to see someone they know and for us it means that we are to a larger extent a part of planning the service they get when they return home [ … ] You get more insight into what has happened and what their needs are further on and it becomes easier to assess their needs when they come home*. (Participant 1, home health care services)

These meetings could thus be regarded as being an important link between the older patient’s situation before and after stays in hospital or intermediate care. The greater involvement of home care services increased the chance that suitable services were given after discharge and that what mattered to the patient was followed up on at home.

However, to fulfill their new roles and responsibilities in the pathway, there was also a perceived need to get to know the older person better or even differently in home care. In line with the PaTH, there was a perceived need to plan ahead and, to a larger degree, be proactive about care, the ultimate goal being to keep the older patients safe at home. HCPs in home care should think more in terms of early detection of symptoms of a worsening chronic condition and should take measures to prevent deterioration in physical function in older care recipients. One participant said the following:

*The culture in our services has to change so that we take more responsibility for our users, so it becomes a mantra that they should be living at home for as long as possible, because I believe that we can do very much to keep them at home, given that they wish to themselves.* (Participant 8, home health care services)

It was emphasized that in the current home care system, the symptoms of a worsening condition were often overlooked because there was a tendency for task orientation in home care, which was precipitated by tight time schedules. The participants were concerned that some HCPs just did their assigned tasks without looking at the totality of the older person’s medical situation. This new way of getting to know the patient resembled the ways HCPs in the hospital and institutions reasoned and worked, for example, with a larger focus on frequent standardized assessments of vital signs and physical function and a greater focus on knowing the diagnoses and medical history of the users. In this respect, home care workers would also appear more professional and knowledgeable, which was perceived to be important to gain trust and increase the patient’s and family’s sense of safety. In conclusion, a central feature of working more *holistically* and in a person-centered way seemed to be combining knowledge from all the HCPs in the different settings in the patient pathway.

#### Ambiguity toward checklists and practice implementation

One important function of the PaTH checklists and the local tailoring of these was to ensure that the things they were discussing and agreeing upon in the QIC would actually be done in practice after the QIC was over. However, despite a general enthusiasm for the checklists’ potential to improve the patient pathways of older people, there was also some skepticism toward these checklists. The checklists were maybe too simplistic of a solution. During one meeting, an improvement team leader said: *The checklists are a symptom and not necessarily a solution*. (Observation note, team leader meeting) Here, the need for the checklists was a symptom of the complexity of the current transitional care system, and this was not solvable through checklists alone. Likewise, scarce resources was a salient topic of discussion. Furthermore, there was an ambiguity in the findings because checklists were believed to make work more systematic and predictable; however, at the same time, the very unpredictable nature of the work with older people, as well as contextual constraints, could lead to a lack of prioritizing of the checklists:

*But then something happens and you don’t have the time or if you are able to make the assessment, the technical equipment isn’t in place and you have to go back to the office and write it on the computer, so then it is down-prioritized and the assessment form is just left somewhere.* (Participant 7, home health care services)

Many had experienced that the current checklists were not being used properly in practice but nonetheless believed that the new tailored checklists would be used in the subsequent scaled-up implementation if the HCPs only understood the purpose of them. One said the following:

*I don’t think the implementation of the checklists will be easy, but I think if we manage to make people understand the value of them, and why we use them. It is to improve the users’ lives.* (Participant 3, home health care services)

The participants expressed appreciation of the bottom-up approach toward quality improvement because it facilitated ownership and local anchoring of the improvement work. However, it also seemed to create a great deal of uncertainty regarding mandates and a blurring of responsibility. One important reason was the lack of power the participants had to make decisions regarding the actual implementation of their chosen quality improvement measures. During a meeting, one participant said: *We can take more responsibility and identify challenges, but they have to be solved at a higher level* (Observation note, team leader meeting). Some expressed doubts regarding the possibilities of creating *real* change in older people’s pathways as a result of the QIC. During a meeting, one participant said: *I hope we haven’t spent millions and done lots of work just to see it run out in the sand* (Observation note, improvement team meeting).

Leadership support from both front-line managers and leaders higher up in the hierarchy was deemed crucial to achieve more person-centered patient pathways in the municipality. This involved both the power to make decisions regarding practice implementation and to allocate the appropriate resources. Thus, improvements at the front-line level were perceived to be strongly connected with improvements at the organizational level.

## Discussion

The aim of the current study was to explore HCPs’ perceptions and experiences of what is important to achieve more person-centered patient pathways for older people by following the work of a QIC. The following discussion focuses on how the results relate to previous research on central concepts relevant to person-centered patient pathways for older people, especially the crossing of knowledge boundaries.

### Crossing knowledge boundaries with the help of the older patient’s perspective

A central aspect of the QIC was knowledge sharing, which is recognized as an important strategy for overcoming knowledge boundaries and thus an important aspect of high quality transitional care [8, 47–50]. However, previous research on transitional care interventions tends to focus more on knowledge sharing among HCPs [18, 45, 48] and less on the potential benefits of the patients’ knowledge in such knowledge sharing processes [1, 26]. In the current study, the HCPs gained access to the older patient’s perspective on the pathway through patient interviews, patient journey mapping and collaboration with user representatives. Previous research [77] suggests that HCPs tend to believe that they already know what matters to patients, but when they engage in knowledge sharing processes with patients they are surprised by what they learn. In line with this, our findings show how the HCPs experienced discovering something new when the meaning of the patient pathways from the older patient’s perspective became known. Patient journey mapping seemed to bring about a new, more shared understanding of the *whole* patient pathway for older people in the municipality. In line with these findings, the potential for patient stories and patient journeys to improve quality have been highlighted in previous transitional care research [8, 26, 40]. HCPs from different settings are limited in their view of the whole patient pathway by physical, organizational, cultural, and knowledge boundaries and this may lead to fragmentation of care [8, 26, 40]. As our findings confirm, HCPs’ knowledge of the patient pathway is often confined to what goes on in their own setting and in relation to their understanding of the patient. Scott [26] emphasizes how patients who go through care transitions are in a unique position to make available aspects of care that are otherwise unavailable to clinicians, such as the whole continuum of care service. Our findings are thus in line with previous research which suggests that a larger degree of system thinking among HCPs is important to improve transitional care quality [8, 10] and highlights the role that knowledge sharing with older patients may have in this.

Waring et al. [47, 48] emphasize that knowledge sharing is more than the communication of information. It entails the exchange and use of meanings, practices, and taken-for-granted assumptions between different groups to create shared understandings and collaborative practices [49, 50]. Important in this respect is the concept of *epistemic communities,* which refers to the social groups that produce, use, and value knowledge in different ways [78–80]. Building on this it can be argued that the older patients and HCPs have different *ways of knowing* the patient pathway and thus represent different epistemic communities. Furthermore these ways of knowing need to be reconciled to achieve more person-centered patient pathways for older people. Whilst previous transitional care research tends to give older patients a more passive role as recipients of information, education and care [1, 15, 16, 21, 29] our findings imply that giving older people a more active role in knowledge sharing activities is valuable.

Similarly the different *ways of knowing* the patient among HCPs from different settings may illustrate how they also come from different epistemic communities [48]. The participants in the current study emphasized that learning about and from each other was important for pathway improvement, especially regarding how to assess and get to know the older patient. Ward et al. [81] argue that collaborating groups of HCPs, such as the current QIC, not only share and exchange knowledge, but also coproduce new knowledge through active engagement in perceived shared problems. Through discussions about bottlenecks and issues in the pathways, the participants were able to identify knowledge boundaries and mediate them to develop a more shared understanding of what is important to make the patient pathways more person-centered. Establishing a common language, common meaning, and common purpose was highlighted. This can be referred to as knowledge “bonding” and is suggested to be important to improve the quality of care transitions [82]. In the current study, the person- centered perspective represented by the patient journey and WMTY seemed to play an important part in facilitating this knowledge bonding. The participants started seeing each other more like “colleagues working around the user.”

Consistent with previous studies [45, 58, 83], there seemed to be an expansion of the perspectives of the different collaborating HCPs, making it possible to see a more comprehensive or *holistic* way of working. Holistic could here be understood as a more biopsychosocial approach to care [84], which is a central principle of PCC [30]. There seemed to be both an expanded awareness of older peoples’ home situations among hospital and intermediate care workers and the perception that home care providers should focus more on disease management and physical function. This concurs with an increased emphasis on home care and more proactive care, which is emphasized in the PaTH and other well-known transitional care models in the literature [1, 14, 16, 21, 22, 37]. Furthermore, this aspect from the findings can be connected to the concept of *knowing the patient* [32]. The examination of this process in clinical settings shows how nurses acquire context-specific knowledge about the patient, which is central to skilled clinical judgment. Knowing the patient causes certain aspects of the situation with a patient to stand out as important when compared with other aspects. Nurses can use this knowledge to make qualitative distinctions, comparing the current situation to known typical situations and then recognizing the relevant changes and early warning of clinical problems, eventually resulting in better informed clinical judgments [85]. Hence, by *knowing the patient* and being properly involved during care, it is easier to detect when things are “out of the ordinary” for the patient and make clinical judgments based on this [33]. This may be particularly important with older people, whose health status may change quickly and pathways are unpredictable [1, 2, 20, 27], which was also seen as a challenge by the HCPs in this study. Conversely, the consequence of *not* knowing the patient is detrimental to care [33] and may lead to adverse events. This points to the issue of task orientation in home care, which was brought up in the findings.

### Home care providers as knowledge brokers during the older patient’s journey

To facilitate the crossing of knowledge boundaries, the literature refers to knowledge *brokers*, which are the more or less formal roles people take to pass on knowledge and information between actors or epistemic communities [31, 48, 79]. An important finding in this respect was the emphasis on the unique knowledge of the HCPs who were accustomed to working in the older person’s home and thus worked close to the older person’s life context. Consistent with the findings of Røsstad et al. [57], who investigated the development of the PaTH, home care providers’ knowledge seemed to *gain ground* among collaborating HCPs in the current study. This implies a potential turn toward focusing more on the older patient’s larger life context and their possibility to self-manage and be able to live in their own home. Moreover, this would be consistent with the concept of person-centered patient pathways where focusing on the life context of the patient is key [1, 25].

Dyrstad and Storm [31] discuss how in the context of hospital admission and discharge, the next of kin brings valuable information about the older persons’ medication and physical condition (what is normal and not), thus assuming the role of knowledge broker during transitions. Our findings suggest that because they take on an important function in providing information about the patient’s situation, providers of home health care services may also be important knowledge brokers in the current context. The importance of provision of interpersonal continuity of care by a trusted HCP is in line with older people’s preferences as highlighted in recent reviews [12, 29]. Previous interventions such as Naylor’s [20] and Coleman’s [27] transitional care models employ designated nurses as care coordinators or coaches, but these inhabit more formal roles and are often based in the hospital [16, 19]. Given the increased focus on the role of community- and home care in transitional care of older people [2, 6, 14, 19, 22], a potential for a more formal coordinating role for home care providers in the current context is arguably an implication of our findings.

### Crossing knowledge boundaries with the help of checklists and the WMTY question

The literature refers to *boundary concepts* and *boundary objects,* which can be used to facilitate boundary crossing between actors and distinct social groups [36, 83, 86, 87].

These concepts and objects share the common attribute of being loose enough to encompass the meanings and values of diverse actors but at the same time concrete enough for these actors to be able to tie them to their own specific social world while maintaining their own identity [86, 87]. One of the strengths of standardized CPs, such as the PaTH, which has been highlighted in previous research, is their function as a boundary object that facilitates collaboration among HCPs [36, 83, 87]. We found that the PaTH had a similar function in the current context. In many improvement strategies, knowledge is understood as an explicit, abstract, and tangible resource that can be accessed and shared with others in the form of documents or evidence. This contrasts with the idea that knowledge or know-how is often experiential, tacit, and situated in practice; it is not a “thing” that a community “has,” but rather, it is what they “do” and who they “are” [48]. In line with this, the PaTH checklists were presented as explicit and tangible documents to be shared among the participants, but as the participants worked on them, more experiential and taken-for-granted knowledge came to the surface. Hence, the checklists facilitated a knowledge sharing process that seemed to be just as important, if not more important, than the actual checklists. The participants’ skepticism toward the checklists *in use* is supported by previous research that shows that when checklists meet the complex and *messy* realities of practice contexts, they are often abandoned [36, 45, 58, 88]. Because of the contextual complexity of transitional care, standardized interventions such as checklists may fail because they are based on a too technical-functional view of service organization [79, 82]. Several authors [1, 12, 18, 88] emphasize that holistic and multilevel strategies are needed to make care transitions more person-centered, which is also reiterated by our findings.

Additionally, the WMTY question which was incorporated into the checklists seemed to work as a boundary concept [86] in the current context. The participants emphasized that the WMTY question had different meanings in different contexts, but it also seemed to signify the same thing to everyone: a tool to achieve more person-centered patient pathways. Löwy [86] illustrates the importance of loose concepts in the construction of interdisciplinary alliances in science. Similarly, WMTY seemed to facilitate the QIC participants’ alliance around a common person-centered agenda; around *what matters* to the older patient*.* In this way, our findings are in line with previous research based on both patients’ and HCPs’ experiences [1, 42, 56, 89] implying that *what matters* to the older patient can improve transitional care quality by serving as a “bridge” between the different transitional care settings.

### A call for top-down support

The main challenge outlined by the participants seemed to be moving from working on the ideal patient pathway and the local tailoring of checklists to the actual implementation of their improvement measures into sustainable practice changes. Previous research shows how PCC is often embraced by HCPs at the principle level, but because of contextual constraints, such as scarce resources, organizational frames, and leadership issues, implementation into practice is challenging [43, 44]. Taking into account the significance of spending time with older patients to get to know them [33], one cannot overlook the significance of appropriate resource allocation, especially in primary care, to achieve person-centered patient pathways for older people. Supported by previous research [1, 42, 44, 90], appropriate support from the organizational and political levels is important.

### Strengths and limitations

The strengths of the current study include a complementary data collection method [60] and the use of a well-known and structured form of analysis [73]. The complementary data collection allowed for informal member checking, but formal member checking was not performed and could be regarded as a limitation [60]. The first author was immersed in the field and had the challenge of balancing closeness and distance as a participant observer [61, 72]. However, the inclusion of several researchers in a reflexive analytical process should enhance a study’s trustworthiness [60, 61]. It should be noted that the participants were chosen by their superior to take part in the QIC. This might mean that they had opinions and attitudes similar to their leaders or that they may have felt compelled to answer interview questions in ways that might be favorable to their employer. However, the participants were assured about confidentiality and the independence of the research project in relation to the QIC before interviews. Also, the first author experienced them as being reflective and critical to the interview topics. There was a risk of social desirability bias [91], both in the interviews and observations. However, combining the two methods reduced this bias to some extent [69], and we have also accounted for the variety of the HCPs’ experiences and perceptions in the results. The theme concerning the importance of home care is undeniably influenced by the context and the sample of interviewed participants who were mainly home care workers. However this was also a salient theme among participants who worked in other settings and was evident both during observation and interviews. Qualitative studies do not aim for generalizability [61, 75]; however, we believe that the findings may have important implications for stakeholders and researchers with an interest in older persons’ patient pathways. We have included important contextual information to clarify the potential transferability of the results [61].

### Practice implications

The findings can assist stakeholders in understanding factors important to practicing person-centered transitional care for older people. Our findings reiterate the need for situated encounters to enable knowledge sharing between collaborating partners and letting the older patient’s perspective on the pathway play a central role. Knowledge sharing with patients, such as interviews, mapping out the patient’s journey and working with user representatives seems to facilitate the inclusion of the patient’s perspective in care. Furthermore, based on this, HCPs may develop a shared understanding of the *whole* patient pathway, which points towards less fragmented care. The ability to understand the larger transitional care system around the patient and to work in less of a task-oriented manner was highlighted. Moreover, regarding documentation, including *what matters* to the patient in discharge notes and hand-over reports may facilitate a common person-centered focus across settings. Appearing as an informed unit to the patient was emphasized. Person-centered patient pathways seem to rely on *knowing the patient,* which also points towards more proactive care. This in turn requires appropriate time and resources. A bottom up approach to improving transitional care seems to be appreciated by HCPs, but may create some uncertainty with regards to mandate and requires continuous leadership support. Formalizing relationships and establishing permanent structural arrangements that obligate and support person-centered knowledge sharing collaboration seems crucial.

Future research should clarify whether the current pathway can be improved by assigning home care providers a more formal coordinating or navigating role such as the ones used in other transitional care models. Moreover, a topic for future research is a further exploration of the more direct influences that a person-centered knowledge sharing transitional care intervention may have on daily transitional care practices for older people. The next steps for the current QIC would be implementation research around the use of the program across the multiple contexts and with the older person at the heart of the work.

## Conclusion

The importance of getting a more holistic understanding of the patient pathway by merging different ways of assessing the needs of older people and reconciling different *ways of knowing* was highlighted as important to achieve more person-centered patient pathways for older people in the municipality. The WMTY question operated as a boundary concept and was perceived as an important tool to keep a person-centered focus across all settings and providers in the pathway. Placing the older patient’s journey and *what matters* to the patient at the heart of collaborative quality improvement processes may contribute to boundary crossing both between HCPs and between HCPs and their older patients. This may provide a potential basis for a shared understanding of the *whole* patient pathway and assuming a shared responsibility for quality improvement. Moreover, the findings reiterate implementation of person-centered patient pathways as a complex task in which HCPs need support from their leaders and organizations. Several of the findings could be explored further from a patient perspective and/or by observing actual practice at the front-line. In particular, the role of home care providers in the patient pathways of older people warrants further exploration.

## Data Availability

The datasets generated and analyzed during the current study are not publicly available because of the terms of the data collection approval but parts of the data can be made available from the corresponding author upon reasonable request.

## Abbreviations

HCP: Health care provider
QIC: Quality improvement collaborative
CP: Care pathway
WMTY: *What matters to you?*
PCC: Patient- or person-centered care
PaTH: Patient Trajectory for Home-dwelling elders
GP: General practitioner
COREQ: Consolidated criteria for reporting qualitative research
